# Season Dependent Changes in the Expression of Protein Kinase C Isoenzymes in a Female Patient with Systemic Lupus Erythematosus

**DOI:** 10.1007/s12253-019-00591-7

**Published:** 2019-02-04

**Authors:** Sándor Sipka, Boglárka Brugós, Gabriella Czifra, Zoltán Griger, Norbert Balogh, Tünde Tarr, Gábor Papp, Tamás Bíró, Margit Zeher

**Affiliations:** 1 Division of Clinical Immunology, Faculty of Medicine, University of Debrecen, Móricz Zs. str. 22, Debrecen, H-4032 Hungary; 2 Department of Physiology, Faculty of Medicine, University of Debrecen, Nagyerdei blvd, 98, Debrecen, H-4032 Hungary

**Keywords:** European white women, Peripheral blood mononuclear cells (PBMC), Protein kinase C (PKC), Season dependence, Systemic lupus erythematosus (SLE)

## Abstract

We aimed to answer the question whether the decreased expression of protein kinase C (PKC) isoenzymes in the peripheral blood mononuclear cells (PBMC) of patients with systemic lupus erythematosus (SLE) is inherited or not. For this reason we examined the expression of PKC isoenzymes in a European white girl with acute SLE and in her healthy mother and father simultaneously in summer and winter during one year using western blotting and densitometry. We found that in the father the expression of PKC isoenzymes did not differ from that of eight healthy controls included women and men. However, in the “SLE-free” mother and in the patient arrived in July with acute symptoms of lupus, the expression of PKC isoenzymes showed a season dependent undulation in parallel. Namely, in summer the expression values were significantly lower, in winter they were significantly higher than those in the controls. Thus, the decreased expression of PKC isoenzymes in the PBMC of SLE patient is not a disease specific marker; it appears also in her lupus free mother. This phenomenon may be due to a season dependent female genetic background. However, the low PKC levels in summer can still decrease further the low production of IL-2 in T cells of lupus patients augmenting the existing AP-1 defects. This is the first report on the season and female dependent inherited changing of PKC expression in a European white patient with SLE and her mother. Further studies are needed to confirm these findings in other populations.

## Introduction

Earlier, our group described the decreased expression of several protein kinase C (PKC) isoenzymes both in monocytes (ε, δ, η, ξ) and T cells (β, ε, δ, η, θ, ξ) of systemic lupus erythematosus (SLE) patients consisting of white European women in 90%; additionally, we also reported the elevating and restoring effects of glucocorticosteroid (GCS) treatment in vivo and in vitro [1]. However, it remained obscure whether these PKC impairments were inherited or acquired. To answer these questions, we have got an exceptional chance to investigate a European white family where the expression of PKC isoenzymes could be tested simultaneously in the peripheral blood mononuclear cells (PBMC) of healthy parents and the daughter with fresh acute symptoms of severe SLE in different seasons during one year. Their results were compared to the group of 8 healthy European white control subjects.

## Materials and Methods

### The Patient and her Parents

The 20-year-old girl arrived in July (summer I) with some purpuras on the leg, maculopapular rash and livedo reticularis, oedema around the eyes and Raynaud’s phenomenon. White blood cell count was 3530 cells/mm^3^, platelet count was 79,000 cells/mm^3^. The autoantibody profile showed anti-nuclear antibody (ANA) positivity in 1:160 serum dilution (homogenous pattern), and an increased level of anti-dsDNA antibodies (74.1 versus 18 IU/ml). In addition, anti-platelet antibody positivity, lower complement factor 4 level (0.09 g/l), and anti-Epstein-Barr virus serum IgG positivity were demonstrated. Liver and renal tests were normal without proteinuria. The SLE was diagnosed according to the criteria of SLICC group based on the presence of photosensitivity, maculopapular rash, leukopenia, thrombocytopenia, ANA, anti-dsDNA positivity, representing an SLE Disease Activity Index (SLEDAI) value of 14. The therapy started with 0.5 mg/kg of methylprednisolone (MP) (32 mg in total) and 250 mg of hydroxychloroquine (HC). The dose of MD was gradually reduced to 8 mg until September, however the 250 mg of HC was continued.

The patient’s family history was negative for any autoimmune diseases; the 40-year-old healthy mother was still before menopause and working as hairdresser. The healthy 41-year-old father only with an abnormal glucose tolerance was working as truck driver. The 8-year-old younger brother was also healthy. The family belongs to the European white population.

In September, at the second visit of patient taking MP and HC, the clinical state improved remarkably (SLEDAI was 5) but the platelet cell count was still low (20,000 cells/mm^3^) without any bleeding complications. Therefore, the dose of MP was increased, HC remained and additionally azathioprine (AZA) was introduced (48 mg of MP + 250 mg of HC + 150 mg of AZA). In December (in winter), at the third visit, the dose of MP was reduced to 8 mg, HC and AZA therapies were continued. The platelet count was already elevated (112,000 cells/mm^3^) and SLEDAI was 8. In July, at the fourth visit (in summer II), the clinical state improved further (SLEDAI was 3), the platelet count became 196,000 cells/mm^3^ and 8 mg of MP was continued together with 250 mg of HC, while AZA treatment was stopped. The laboratory tests still showed ANA and anti-dsDNA positivity (60.9 IU/ml). Now, the patient has no clinical symptoms and receives 8 mg MP and 250 mg HC as maintenance therapy.

In December and the following July (summer II), the serum levels of three thyroid hormones free thyroxine (FT4), free triiodine-thyronine (FT3) and thyroid stimulating hormone (TSH) were measured in the patient.

All three members of family gave a written consent confirming their readiness to take part in this study and permit to publish the results.

### The Group of Healthy Controls

We enrolled 6 women (range of age: 21–43 years) and 2 men (22 and 42 years old) who were chosen actually at the various visit times of patient and mother.

### Determination of PKC Isoenzymes in PBMC

PBMC were isolated by Ficoll (Sigma-Aldrich, USA) gradient centrifugation. The expression of PKC isoenzymes were evaluated using western blotting and densitometry. Human β-actin served as an endogenous internal control.

### Statistical Analysis

Using SPSS 20 program, the numeric data of densitometry were analysed and compared by Wilcoxon Signed-Rank (non-parametric) test. Changes representing values of *p* < 0.05 were regarded as statistically significant.

## Results

### Season Dependent Changes in the Expression of PKC Isoenzymes in PBMC of SLE Patient, in her Parents and Three Healthy Controls

**Fig. 1:**
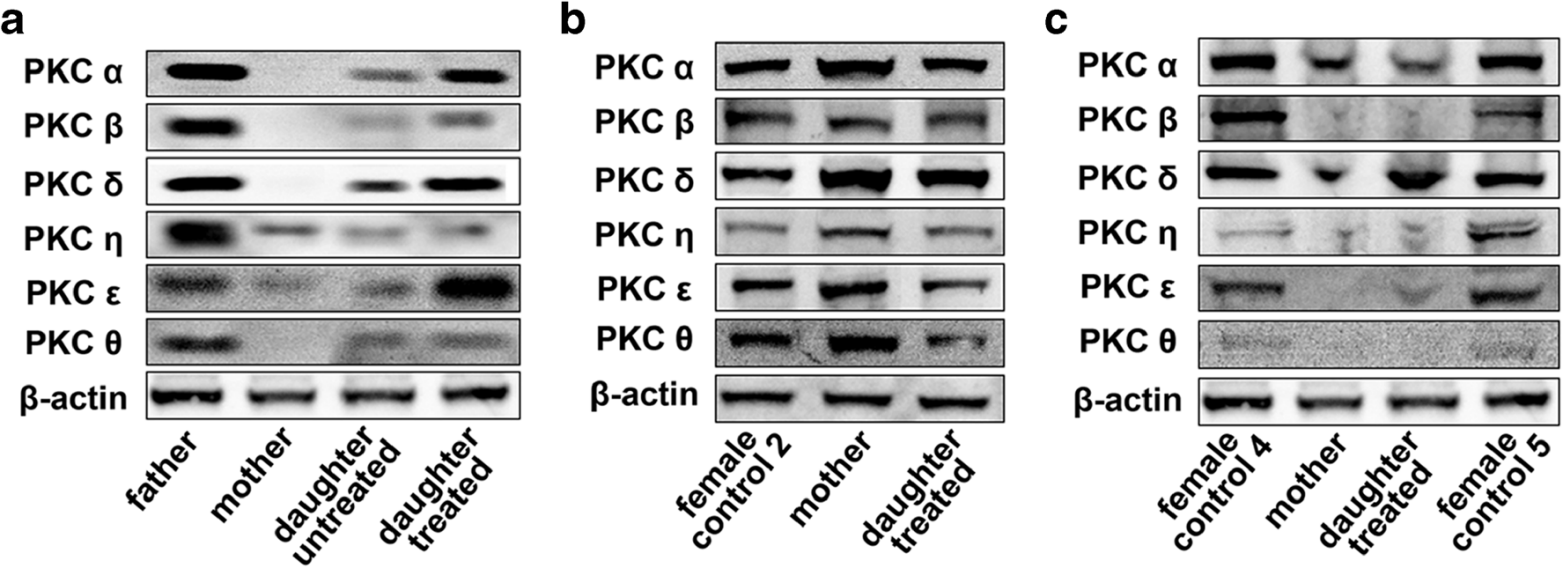
The representative images of western-blotting analysis on the expression of PKC isoenzymes in the peripheral blood mononuclear cells of (**a**) patient with SLE and in the healthy parents tested in summer I; (**b**) patient with SLE, in the mother and in a healthy control tested in winter and (**c**) patient with SLE, in the mother and two healthy controls tested in summer II

Figure 1a demonstrates that at the first visit of the whole family in July (summer I), the expression of all PKC isoforms (α, β, δ, ε, η, θ) in the PBMC of healthy father was equally strong, and did not differ from the intensity of β-actin. On the other hand, the expression of isoenzymes was decreased remarkably both in the lupus patient and her healthy mother compared to the values of β-actin and the father’s results. For September, the intensive MP therapy elevated and almost restored the PKC levels, and resulted in a great improvement in the clinical state of patient.

However, in December (winter), both in the patient (treated with MP, HC and AZA) and her “SLE-free” healthy mother without any medication, the expression of the PKC isoenzymes elevated remarkably compared to the values of “female control 1” (28-year-old European white woman) (Fig. 1b).

Whereas in the following summer (summer II), the PKC values of both the patient (taking MP, HC and AZA) and her mother were lower again than those of “female control 4” (22-year-old European white woman) and “female control 5” (41-year-old European white woman) (Fig. 1c).

### Numeric Data of Densitometry on the Expression of PKC Isoenzymes in PBMC of the Family and Healthy Controls

Table 1 presents all the numeric data of densitometry on the expression of PKC isoenzymes in PBMC of the family followed up for one year. In the data of the first visit in July (summer I), then in September, the values of father’s densitometry were regarded as 1.0 and the data of “mother” and “patient” were compared to them and to their internal control, β-actin. Later, in the further simultaneous studies on the cells of “mother” and “patient”, the data of densitometry were statistically compared to the average of 8 subjects forming the “group of healthy controls”. It was a striking surprise at the first visit that in the PBMC of healthy mother, the expression of each PKC isoenzymes showed not only significantly lower values than those in the healthy father and healthy controls (0.09 + 0.23 versus 1.04 + 0.24; *p* < 0.05), but they were even lower than those in her sick daughter (0.09 + 0.23 versus 0.33 + 0.27). In the patient, we recognized a similar pattern what we published earlier [1] showing significant decrease in the expression of various PKC isoenzymes (0.33 + 0.27 versus 1.04 + 0.27; *p* < 0.05), and the significant, almost total restoring effect of MP (0.88 + 0.64 versus 0.33 + 0.27 *p* < 0.05). However, at the following visit in December (winter), each PKC level, both in the mother and patient, increased significantly compared to the values of healthy controls (mother: 1.53 + 0.69; patient: 1.48 + 0.88; versus controls: 1.04 + 0.27; *p* < 0.05). On the other hand, in the following July (summer II), the PKC levels were significantly decreased again in both subjects, in the mother: 0.43 + 0.36; in the patient (taking 8 mg of GCS + HC): 0.58 + 0.48 versus in the controls: 1.04 + 0.27 (p < 0.05).

**Table 1:** Numeric data of densitometry on the expression of PKC isoenzymes tested by western-blotting analysis in the peripheral blood mononuclear cells of family members followed up for one year and in the healthy controls

Seasons	PKC	Father	Mother	Daughter SLE	Daughter SLE treated	Controls (*n* = 8)
Summer 1	α	1.00	0.00	0.27	0.90	1.11
	β	1.00	0.00	0.07	0.50	1.11
	δ	1.00	0.00	0.66	0.98	0.88
	η	1.00	0.35	0.12	0.21	0.79
	ε	1.00	0.20	0.61	2.20	1.51
	θ	1.00	0.00	0.29	0.58	0.84
Average ± SD		„A” 1.00	„B″ 0.09 ± 0.23	„C″ 0.33 ± 0.27	„D” 0.89 ± 0.64	„E” 1.04 ± 0.27
Winter	α		2.60		2.80	1.11
	β		1.44		1.20	1.11
	δ		1.70		1.63	0.88
	η		1.54		2.10	1.51
	ε		1.05		0.66	0.79
	θ		0.98		0.50	0.84
Average ± SD			„F″ 1.56 ± 0.59		„G” 1.48 ± 0.88	„E” 1.04 ± 0.27
Summer 2	α		0.63		0.85	1.11
	β		0.13		0.16	1.11
	δ		0.90		1.34	0.88
	η		0.72		0.74	1.51
	ε		0.09		0.30	0.79
	θ		0.11		0.09	0.84
Average ± SD			„H″ 0.43 ± 0.36		„I″ 0.58 ± 0.48	„E” 1.04 ± 0.27

Significant differences (p < 0.05) were found between B-E, C-E, C-D, F-E, G-E, H-E and I-E values

### Changes in SLEDAI Values, Serum Levels of Thyroid Hormones along with the MP Treatment of SLE Patient during the Study

In Table 2, we summarized the changes in SLEDAI values of patient and doses of MP with other immunosuppressive drugs. Moreover, we also compared the serum levels of three thyroid hormones in December and following July. The three hormones, TSH, FT4 and FT3 regulating the seasonal adaptation, showed slightly higher concentrations in winter than in summer suggesting a moderate suppression of gonadotropin secretion. However, in summer the situation was inversed, the decreased thyroid hormone levels might involve an elevated gonadotropin secretion [2].

**Table 2:** Changes in SLEDAI values, serum levels of thyroid hormones and doses of glucocorticosteroids at the SLE patient during the study

Tests/Treatments	July I	September	December	July II
SLEDAI	14	5	8	3
TSH [mU/l]	n.t.	n.t.	3.6	2.3
FT4 [pmol/l]	n.t.	n.t.	17.2	14.2
FT3 [pmol/l]	n.t.	n.t.	6.7	5.5
MP [mg] + other treatments	none	8 + HC	8 + HC + AZA	8 + HC

*AZA*, azathioprin; *HC*, hydroxychloroquine; *FT3*, free triiodide thyronine; *FT4*, free thyroxine; *MP*, methylprednisolone; *SLEDAI*, SLE disease activity index; *TSH*, thyroid stimulating hormone; n.t, not tested

## Discussion

This is the first report on the “season and GCS and thyroid hormone dependent changing of PKC isoenzymes as a special phenomenon of female genetic background” in the mononuclear cells of a European white female patient with newly diagnosed acute SLE and in her lupus free mother. Nevertheless, this special genetic background is not sufficient alone to cause SLE yet. Still other pathologic hits may be also required to induce and complete the disease. However, presumably in *white female patients with lupus in summer,* the decreased expression of PKC isoenzymes can lead to a state diminishing further the basically low production of interleukin (IL)-2 in T cells augmenting the existing defect in the function of activating protein-1 (AP-1) via the extracellular signal-regulated kinase (ERK)1/2 [3, 4] and other AP-1 related pathways [5, 6], or the weak effect of PKC θ in alternative splicing processes regulating gene expressions during T cell activation [7]. In order to verify the wider existence and distribution of this phenomenon, still further comparisons are needed in different genders [8, 9], countries and continents [10]. In addition, further genetic mapping of this family would be of worth excluding any splicing or other variations.
